# Structural and Optical Properties of Nickel-Doped Zinc Sulfide

**DOI:** 10.3390/nano14191599

**Published:** 2024-10-03

**Authors:** Sultan Alhassan, Alhulw H. Alshammari, Satam Alotibi, Khulaif Alshammari, W. S. Mohamed, N. M. A. Hadia

**Affiliations:** 1 Department of Physics, College of Science, Jouf University, Sakaka 72341, Saudi Arabia; ahalshammari@ju.edu.sa (A.H.A.); knnalshammari@ju.edu.sa (K.A.); wsmahmed@ju.edu.sa (W.S.M.); nmhadia@ju.edu.sa (N.M.A.H.); 2 Department of Physics, College of Science and Humanities in Al-Kharj, Prince Sattam bin Abdulaziz University, Al-Kharj 11942, Saudi Arabia; sf.alotibi@psau.edu.sa

**Keywords:** ZnS nanoparticles, Ni-doped ZnS, hydrothermal synthesis, optical properties, nanostructures

## Abstract

In this study, undoped and Ni-doped ZnS nanoparticles were fabricated using a hydrothermal method to explore their structural, optical, and surface properties. X-ray diffraction (XRD) analysis confirmed the cubic crystal structure of ZnS, with the successful incorporation of Ni ions at various doping levels (2%, 4%, 6%, and 8%) without disrupting the overall lattice configuration. The average particle size for undoped ZnS was found to be 5.27 nm, while the Ni-doped samples exhibited sizes ranging from 5.45 nm to 5.83 nm, with the largest size observed at 6% Ni doping before a reduction at higher concentrations. Fourier-transform infrared (FTIR) spectroscopy identified characteristic Zn–S vibrational bands, with shifts indicating successful Ni incorporation into the ZnS lattice. UV–visible spectroscopy revealed a decrease in the optical band gap from 3.72 eV for undoped ZnS to 3.54 eV for 6% Ni-doped ZnS, demonstrating tunable optical properties due to Ni doping, which could enhance photocatalytic performance under visible light. Scanning electron microscopy (SEM) and energy-dispersive X-ray spectroscopy (EDX) analyses confirmed the uniform distribution of Ni within the ZnS matrix, while X-ray photoelectron spectroscopy (XPS) provided further confirmation of the chemical states of the elements. Ni doping of ZnS nanoparticles alters the surface area and pore structure, optimizing the material’s textural properties for enhanced performance. These findings suggest that Ni-doped ZnS nanoparticles offer promising potential for applications in photocatalysis, optoelectronics, and other fields requiring specific band gap tuning and particle size control.

## 1. Introduction

The various categories of II–VI materials, including bulk, thin-film, microcrystalline, and nanostructured, consist of a vast array of distinct compounds [1,2,3]. These compounds exist in an enormous range of cationic and anionic forms, as well as a wide variety of surface configurations. Modifications in these domains can significantly impact the electrical and optical characteristics of the materials [4,5,6]. In order to facilitate the modification of these characteristics in systems tailored for particular optoelectronic or photonic purposes, it may be necessary to precisely adjust the chemical composition, dimensions, and microstructure. There are multiple methods to create the desired property alteration, depending on its specific nature. In certain devices like switching or laser devices, the local variation in the electrical state may be the sole factor of significance. Metal sulfides, such as ZnS, MoS_2_, CdS, and WS_2_, have emerged as critical materials in energy-related implementations due to their unique electronic, optical, and catalytic properties. These materials are particularly attractive for use in energy-conversion and storage technologies, including photocatalysis, electrocatalysis, and battery electrodes. In the context of photocatalysis, metal sulfides like CdS and ZnS are known for their ability to absorb visible light and facilitate charge separation, making them highly effective for hydrogen evolution reactions (HERs) and overall water splitting. MoS_2_ and WS_2_, on the other hand, are widely recognized for their excellent catalytic activity in HERs due to their layered structures, which provide abundant active sites for hydrogen adsorption and promote efficient electron transfer. Additionally, the tunable band gaps and high surface area of these sulfides enhance their efficiency in changing solar into chemical energy. In battery applications, metal sulfides are valued for their high theoretical capacity and good conductivity, making them promising candidates for next-generation lithium-ion and sodium-ion batteries. Overall, the versatile properties of metal sulfides position them as vital components in the development of sustainable energy technologies [7]. Zinc sulfide (ZnS) nanoparticles, belonging to the chemical group II-VI, have attracted significant attention due to their potential uses across various sectors [8]. Their relatively large band gap of 3.9 eV makes them highly suitable for use in photocatalysts, cosmetics, luminous materials, and solar cells. ZnS nanoparticles have the capability to adjust the band gap energy by controlling the size of the particles, which is a promising feature. Particle sizes ranging from 2 to 4 nm exhibit an expanded band gap, suggesting an increase in the band gap of the ZnS nanoparticles. This characteristic renders them suitable for the application of UV protection coatings on plastics and other materials. Zinc sulfide (ZnS) can be utilized in the production of ultraviolet (UV) sensors to effectively absorb intense UV radiation. Its non-toxicity and water stability render it a viable substitute for organic UV sensors. Zinc sulfide (ZnS) is recognized for its superior electroluminescent properties in comparison to other sulfides and oxides. This is because when it is electrically stimulated, it releases a luminous and colorless light. III–V semiconductors and organic materials have the ability to emit certain wavelengths of light, which makes them very suitable for the development of white LED technology. Moreover, compared to other sulfides like MoS₂, CdS, and WS₂, ZnS nanoparticles have distinctive electronic and optical properties that are highly tunable through doping, particle size control, and surface modification. This tunability is crucial for enhancing their efficiency in photocatalytic processes, particularly in hydrogen evolution reactions (HERs), where efficient charge separation and transfer are essential. Metal sulfides, including ZnS, have garnered significant attention in recent years due to their ability to reduce overpotentials and improve the kinetics of HERs, a critical reaction in the production of clean hydrogen fuel [9,10]. The introduction also highlights the versatility of ZnS nanoparticles compared to other sulfides. While sulfides like MoS_2_ and CdS are known for their high catalytic activity, they often suffer from the rapid recombination of photogenerated charge carriers and stability issues. ZnS, with its higher stability and adjustable properties through doping, provides a compelling alternative that can be optimized for improved photocatalytic performance. The process of incorporating transition metals into micro- and nano-ZnS has garnered significant interest from numerous researchers [11]. Doped ZnS nanoparticles have superior qualities compared to undoped and bulk ZnS, and these features differ depending on the dopant used. The addition of a dopant can modify various characteristics of ZnS, including its crystal structure, grain development behavior, particle size distribution, stability, optical and electrical conductivity, and magnetic properties. Therefore, doped ZnS was utilized in several fields such as gas sensors, light-emitting diodes, solar cells, transparent conductive films, field-emission displays, varistors, photocatalysts, and lithium batteries. ZnS can be doped with different transition metals at different concentrations using various synthesis processes including chemical co-precipitation, hydrothermal synthesis, and sol–gel procedures, depending on the specific application.

Hydrothermal synthesis is a novel approach for incorporating transition metal ions (TMIs) into ZnS, and there are limited publications on this specific technique. We have successfully produced ZnS of excellent quality by uniformly doping it with TMIs using our method. This was achieved without requiring a separate TMI source and at a comparatively low temperature compared to alternative methods. Hydrothermal synthesis is a technique that is mostly used to crystallize inorganic compounds from watery solutions by exploiting the relationship between solubility and the components involved. The hydrothermal procedure can be employed to synthesize the majority of inorganic crystalline materials [12]. Hydrothermal synthesis is a highly effective method for cultivating inorganic crystalline minerals, owing to its several advantages. Firstly, hydrothermal synthesis sustains elevated ion concentrations. Furthermore, hydrothermal synthesis is the most successful method for incorporating foreign materials’ ions into the crystal lattice due to the extended interaction durations between the components in the complex solution. Hydrothermal processes naturally generate ion dopants due to the concentration gradients between inorganic precursor components. Consequently, it is considered highly important to develop hydrothermal synthesis methods that can effectively incorporate transition metal ions into the ZnS lattice with both high uniformity and concentration. In recent years, the synthesis and characterization of metal-doped ZnS nanoparticles have attracted considerable attention due to their unique structural, optical, and photocatalytic properties. Doping ZnS with transition metals such as Ni has been shown to significantly alter the material’s characteristics, making it more suitable for various applications, including photocatalysis and optoelectronics. For instance, Othman et al., [13] (2020) demonstrated that Ni-doped ZnS nanoparticles synthesized through sonochemical methods exhibited enhanced structural, optical, and photocatalytic properties, highlighting their potential for environmental- and energy-related application. Similarly, Kaur et al. [14] (2015) investigated the photocatalytic efficacy and the photoluminescence of Mn- and Ni-doped ZnS NPs, revealing that doping with these metal ions could effectively modulate the nanoparticles’ optical behavior, thereby improving their functional performance in light-driven processes. Furthermore, the work by Poornaprakash et al. [15] (2017) provided a comprehensive analysis of various characteristics of Co-, Ni-, and Fe-doped ZnS NPs, underscoring the significant impact of metal doping on the material’s multifunctional characteristics. These studies collectively underscore the importance of metal doping in tailoring the properties of ZnS nanoparticles for advanced technological applications, motivating further exploration in this field.

In this work, undoped and Ni-doped ZnS nanoparticles were synthesized via the hydrothermal method. The structural, surface morphology, optical, and photocatalytic properties of the sensitized samples were investigated for potential applications.

## 2. Experimental Section

### 2.1. Synthesis of Zn_1−*x*_Ni_*x*_S Samples

The hydrothermal process was used to synthesize Zn_1−*x*_Ni_*x*_S nanostructures with *x* values of (0, 0.02, 0.04, 0.06, and 0.08). The studies employed analytical-grade chemicals without further purification. Water that underwent a double distillation process was used. The steps involved in making it are as follows: The following substances were mixed to create a solution: stoichiometric zinc chloride (ZnCl_2_), nickel chloride (NiCl_2_), and sodium sulfide (Na_2_S), which were weighed. The mixture was then added to 80 mL of double-distilled water. It was agitated using a magnetic stirrer at room temperature for 3 h until fully dissolved. Afterward, the combined solution was moved into an autoclave made of stainless steel with a 100 mL Teflon liner (with an 80% fill ratio), sealed, and kept in an oven at 180 °C for 16 h. The autoclave was removed once the reaction was complete and allowed to cool to room temperature on its own. After that, the precipitate was washed several times with distilled water and ethanol, and later dried at 100 °C for 10 h. Finally, the Zn_1−*x*_Ni_*x*_S nanostructures were collected and used in further investigations.

### 2.2. Characterization of the Samples

X-ray analysis was performed utilizing an XRD-7000 Shimadzu instrument X-ray diffractometer with Cu Kα radiation (λ = 1.5418 Å). The instrument settings included operating at 40 kV and 40 mA. Scanning electron microscopy was conducted using a Thermo Scientific Quattro instrument. Prior to imaging, samples were affixed onto aluminum stubs using double-sided carbon tape and coated with a thin layer of gold–palladium alloy to enhance conductivity. The microscope was operated at an accelerating voltage of 15 kV, and secondary electron images were obtained at magnifications ranging from 500× to 5000×. Elemental analysis was performed using energy-dispersive X-ray spectroscopy (EDS) with an Oxford Instruments X-MaxN 80T EDX detector attached to the SEM. Fourier-transform infrared (FTIR) analyses were performed with a Shimadzu IR-Tracer 100 spectrometer, acquiring spectra of powdered samples within the 400 to 4000 cm^−1^ range using a KBr pellet, maintaining a resolution of 4 cm^−1^. UV–Vis absorption spectrophotometry was conducted using an Agilent spectrophotometer.

## 3. Results and Discussion

### 3.1. X-ray Diffraction (XRD) Measurements

**Figure 1:**
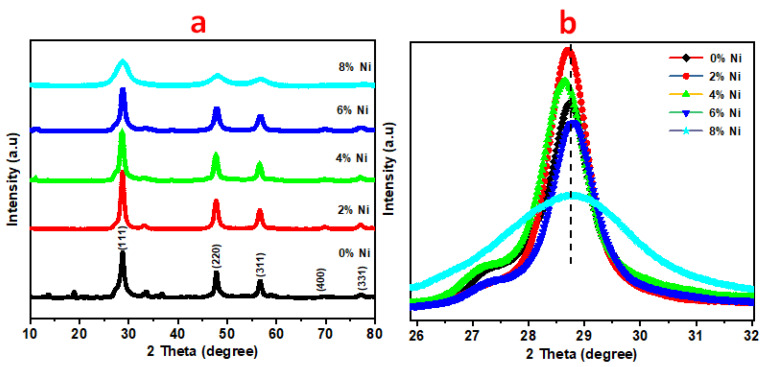
**(a)** XRD patten of pristine and variously Ni-doped ZnS and (**b**) Enlarged XRD patterns of (111) peak.

The XRD technique has been utilized to identify the structure of the nanoparticles; therefore, the XRD measurement is implemented for undoped and doped ZnS with Ni. The XRD patterns 2θ was scanning from 10° to 80°^,^ as depicted in Figure 1. The sample of undoped ZnS showed various peaks, with the main sharp peak located at 28.74°, which corresponds to the (111) plane. The sharp peak indicates the ZnS crystalline structure. Other peaks that were located at 47.96°, 56.77°, 69.77°, and 77.13° are due to the (220), (311), (400), and (331) planes of the cubic phase as reported in (JCPDS 65-0309) [16,17]. In the presence of Ni^2+^, it substituted the Zn^2+^ in the ZnS host lattice. It is noticeable there were no impurity peaks that were evident in the homogenous distribution of Ni and Zn ions. Moreover, the cubic structure of ZnS was not changed when Ni^2+^ replaced Zn^2+^. It is well established that the integration of dopant ions, such as Ni, into the lattice of ZnS can lead to modifications in the lattice parameters, including changes in the lattice constant, which may ultimately affect the crystalline structure. This effect is often concentration-dependent; at lower concentrations, the dopant ions may occupy interstitial or substitutional sites without significantly altering the overall cubic structure. However, as the concentration of dopants increases, lattice distortions can occur due to differences in the ionic radii and bonding characteristics, potentially leading to changes in the lattice constant and, in some cases, even a phase transition.

There are numerous studies that demonstrate how varying dopant levels can influence the structural properties of ZnS nanoparticles [16]. For example, researchers have observed that dopants like Mn in ZnS can cause gradual changes in lattice constants due to substitutional incorporation of these ions [18]. The distortion of the ZnS lattice arises from the size mismatch and different valence states of the dopant ions compared to the host Zn^2^⁺ ions. Therefore, the impact of nickel doping on the structure of ZnS is also expected to vary with the concentration, and this could be further detailed by referencing similar studies. The main peak was located at 2θ = 28.74°, 28.73, 28.66° for the pure ZnS and ZnS/2 and 4 wt% Ni samples, respectively. It is noticeable that the abundance peak was shifted to the greater 2θ when increasing the Ni content up to 4%. Moreover, Table 1 illustrates the changes in interplanar spacing (d values) for ZnS nanoparticles and their Ni-doped counterparts, revealing shifts in the XRD peaks due to Ni incorporation. For the (111) plane, the d-spacing decreases from 3.09257 Å for undoped ZnS to 3.07620 Å at 6% Ni doping, suggesting lattice contraction, before slightly increasing to 3.10657 Å at 8% Ni doping, indicating a minor expansion. The (220) plane shows a gradual increase in d-spacing from 1.89537 Å (undoped) to 1.90367 Å (8% Ni-doped), reflecting lattice distortion. Similarly, the (311) plane exhibits a small decrease from 1.62041 Å (undoped) to 1.61831 Å (2% Ni-doped), followed by an increase to 1.62588 Å at 8% Ni doping. These variations in interplanar spacing confirm that Ni doping induces structural modifications in the ZnS lattice, resulting in lattice strain and distortion. The observed changes align with the substitution of Ni ions into Zn sites, affecting the crystal lattice parameters and indicating a typical pattern of distortion associated with doping in semiconductor materials. The lattice constant values were calculated by Equation (1).

(1) $$d_{hkl}=\frac{a}{\sqrt{h^{2}+k^{2}+l^{2}}}$$

Moreover, that shift may be caused by the ionic substitution since the ionic radii of Ni^2+^ is 0.69 Å while zinc ionic radii is 0.74 Å. However, the most intensive peak was shifted towards lower 2θ to be 28.82° and 28.71° for the ZnS/6 and 8 wt% Ni. This may occur due to the crystal lattice deformation that was induced by the high Ni content in the Zn host lattice.

More analysis is important to show the influence of the presence of Ni on the ZnS by calculating the average crystal size through the Debye–Scherrer equation (see Equation (2)). The crystal size was conducted at the favorable growth orientation plane (111) [16].

(2) $$D=\frac{0.9\lambda }{\beta cos\theta }$$

where the X-ray wavelength and full width at half maximum can be expressed by λ and β. The obtained average crystal size of ZnS is 5.27 nm. However, the average crystal sizes of the ZnS that incorporate 2, 4, 6, and 8 wt% Ni are 5.45, 5.83, 5.71, and 5.60 nm, respectively. The average crystal size was increasing due to the increase in the Ni up to 6% and then decreasing for the sample that had 8% of Ni. The observed trend in the average crystal size of ZnS nanoparticles upon Ni doping can be explained by considering the effects of Ni incorporation on the crystal growth dynamics and the lattice structure of ZnS as the following: (i) When Ni is doped into the ZnS lattice at lower concentrations (2–6%), Ni(II) ions replace some of the Zn^2+^ species in the ZnS crystal structure. This substitution can lead to localized lattice distortion and strain, which initially promotes the reorganization and realignment of ZnS units. This process can lower the overall surface energy of the nanoparticles, thereby facilitating further crystal growth and resulting in an increase in the average crystal size. (ii) At these doping levels, Ni ions may also enhance the crystallinity of ZnS by providing nucleation sites that promote crystal growth. The doping ions can act as a catalyst for the growth process, leading to larger crystal sizes as the Ni content increases up to 6% [19,20]. Furthermore, the average crystal size values have full agreement with the interplanar spacing d_hkl_ and lattice constant a value.

### 3.2. Fourier-Transform Infrared Spectroscopy (FT-IR) Measurements

**Figure 2:**
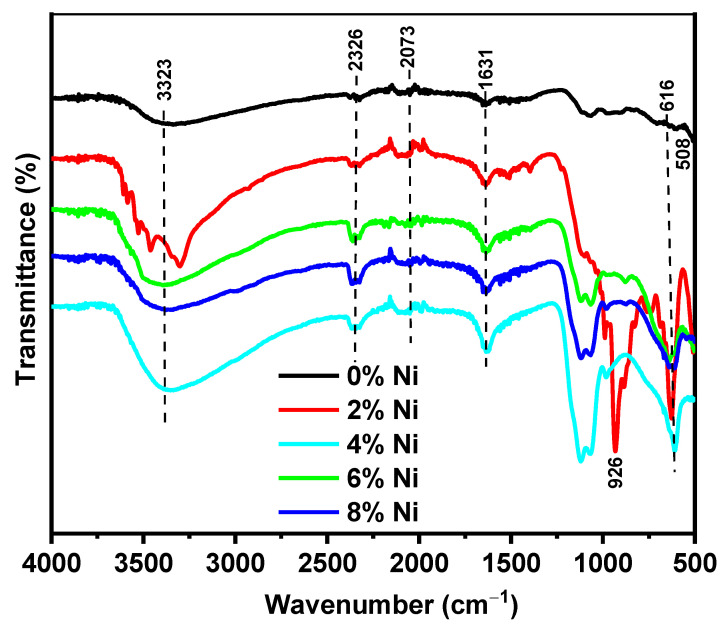
FTIR spectra of pristine and variously Ni-doped ZnS.

FT-IR spectroscopy was employed to investigate the chemical composition and bonding characteristics of both undoped and Ni(II)-doped ZnS nanoparticles (NPs). Spectra were captured in the 500–4000 cm^−1^ range, revealing features indicative of high-quality product formation in the ZnS nanoparticles. The peaks observed at 3323, 2326, 2073, 1631, 501, and 616 cm^−1^ in the FTIR spectra (Figure 2) are noteworthy [17]. The broad and weak peaks at 3323 cm^−1^ are designated to the stretching mode of trace amounts of attached water on the particle surface, likely due to the gyroscopic nature of the fabricated materials. The characteristic peak at 2326 cm^−1^ corresponds to N–H bond stretching modes, while bands at 2073 and 1631 cm^−1^ are associated with the existence of CO_2_ molecules [21]. Notably, peaks at 1053, 617, and 508 cm^−1^ are linked to metal–sulfur bond (Zn–S) vibrations, characteristic of cubic ZnS. In the 2% Ni-doped ZnS sample, the band observed around 926 cm⁻^1^ is typically associated with the bending vibrations of metal–oxygen (M–O) bonds. This peak could indicate the formation of Ni–O bonds due to the incorporation of Ni(II) ions into the ZnS matrix. The presence of this band suggests that some Ni atoms may be forming oxides or interacting with oxygen, which can occur during the doping process or subsequent sample preparation steps. These observed peak values align well with reported values [22], suggesting proper dopant substitution into the host. While the existence of bands at similar wavenumbers suggests successful dopant incorporation, small shifts and alterations in intensities might be related to integration between the dopant and ZnS.

### 3.3. Scanning Electron Microscope (SEM) Measurements

**Figure 3:**
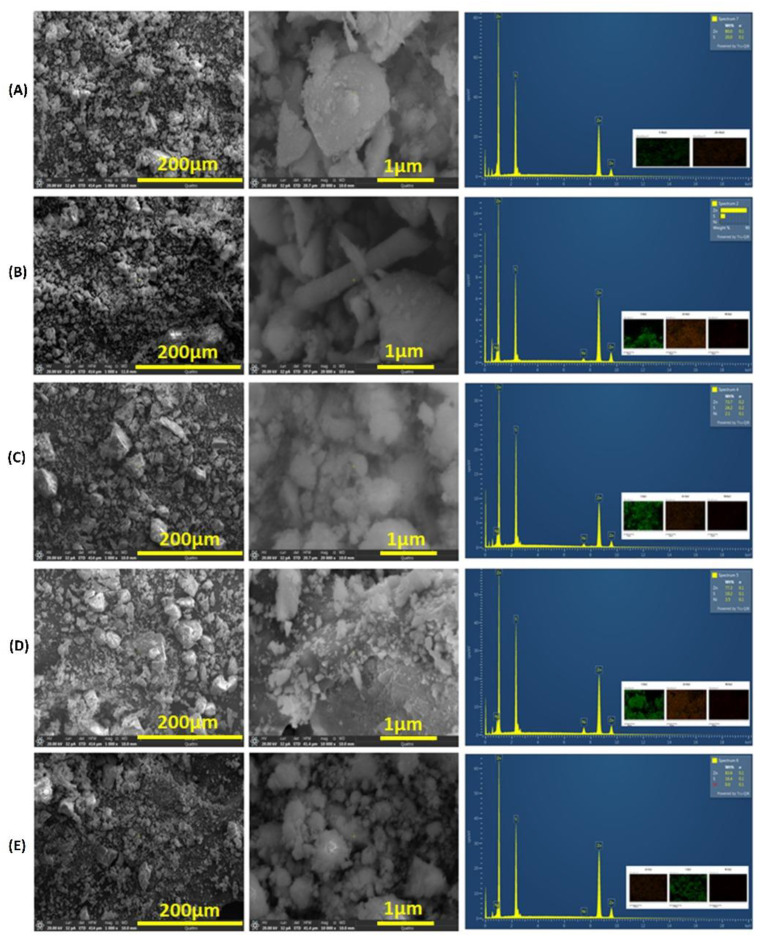
SEM images and EDX spectra (inset is the elemental mapping) of (**A**) pristine ZnS (0% Ni), (**B**) 2% Ni, (**C**) 4% Ni, (**D**) 6% Ni, and (**E**) 8% Ni.

Figure 3 depicts the surface morphology and structural characteristics of pristine ZnS and Ni-doped ZnS nanoparticles with various magnifications. The SEM images reveal that all materials are agglomerated, lacking any morphological characteristics attributable to the dopant. The particles are nearly semi-spherical in shape. This agglomeration is likely due to the nanoparticles’ high relative surface area and the high number of surface atoms with unsaturated coordinations, which have vacant sites. These atoms tend to form bonds with adjacent particles, leading to agglomeration. Additionally, the EDX spectra confirm the presence of zinc, sulfur, and nickel signals, with no other impurities detected within the instrument’s sensitivity limits. Furthermore, the EDX spectra clearly indicate a uniform distribution of elements in the fabricated products, demonstrating that the ZnS nanoparticles are homogeneously doped with Ni. Elemental mapping using energy-dispersive X-ray spectroscopy (EDS) provides further evidence of the elemental distribution and composition of the prepared materials.

### 3.4. Nitrogen Isotherms and Pore Size Distribution

**Figure 4:**
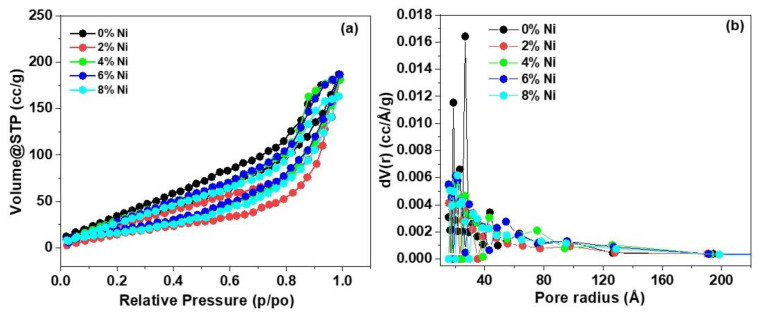
(**a**) N_2_ isotherms and (**b**) pore size distribution of pristine ZnS (0% Ni), 2% Ni, 4% Ni, 6% Ni, and 8% Ni.

The nitrogen adsorption–desorption isotherms (Figure 4a) and pore size distribution (Figure 4b) of the pristine ZnS and Ni-doped ZnS samples reveal insights into the textural properties of the materials. The isotherms exhibit a typical Type-IV pattern with H3-type hysteresis loops, suggesting the existence of mesoporous architecture across all samples. This mesoporosity plays a critical role in enhancing the properties of the materials by providing an increased surface area for the interaction of reactant molecules. Table 2 summarizes the surface area, average pore diameter, and pore volume for pristine ZnS and Ni-doped ZnS samples. The pristine ZnS exhibits the highest surface area (52.66 m^2^/g) and average pore diameter (26.73 Å) among all samples, with a pore volume of 0.2804 cm^3^/g. Upon Ni doping, the surface area shows a non-monotonic trend, initially decreasing at 2% Ni doping (33.78 m^2^/g) and then increasing with higher Ni doping levels up to 6% Ni (44.45 m^2^/g), before slightly decreasing again at 8% Ni (42.94 m^2^/g). This behavior can be attributed to the introduction of Ni ions, which may partially block the pores or cause some agglomeration at lower doping levels (2% Ni), leading to a reduced surface area and pore size. As the Ni concentration increases (up to 6%), the doping likely enhances the pore structure by creating new mesopores or modifying the existing ones, thus increasing the surface area and pore volume. However, at the highest doping level (8% Ni), the surface area decreases slightly, which could be due to further agglomeration or pore collapse, limiting the accessibility of the mesopores. The pore size distribution curves (Figure 4b) further support these findings, where the pristine ZnS shows a broader distribution of pore sizes compared to the Ni-doped samples. The introduction of Ni ions narrows the pore size distribution, with a noticeable shift towards smaller pore diameters. This shift suggests that Ni doping influences the textural properties by modifying the pore structure, which could enhance the adsorption properties and catalytic activity of the ZnS materials.

### 3.5. X-ray Photoelectron Spectroscopy (XPS) Assessment

**Figure 5:**
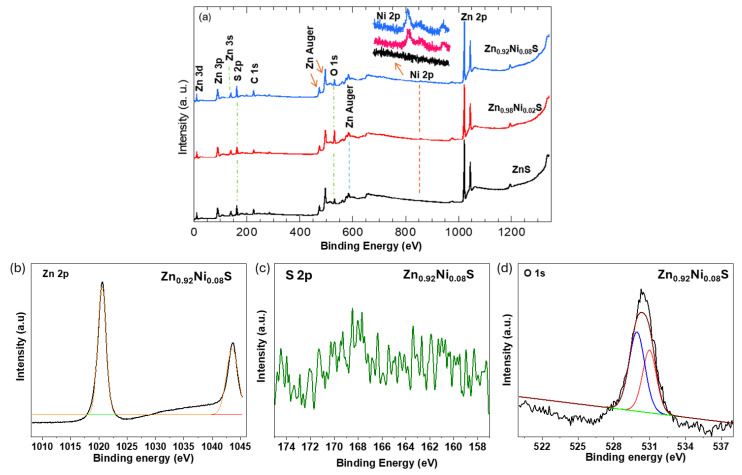
(**a**) XPS survey spectra of the pristine ZnS, Zn_0.98_Ni_0.02_S, and Zn_0.92_Ni_0.08_S, and Deconvoluted XPS spectra of (**b**) Zn 2p, (**c**) S 2p, and (**d**) O 1s of Zn_0.92_Ni_0.08_S.

XP spectroscopy is a versatile technique to verify the chemical composition of the examined samples. Figure 5a presents the wide spectra of the pristine ZnS and Ni-incorporated ZnS samples, namely, ZnS, Zn_0.98_Ni_0.02_S, and Zn_0.92_Ni_0.08_S. The survey spectra of the pristine ZnS clearly exhibit a doublet sharp peak at 1044.3 and 1021.2 eV that is related to Zn 2p1/2 and Zn 2p3/2, respectively. Other tiny peaks corresponding to Zn 3d, Zn 3p, and Zn 3s Zn Augers can also be observed on the survey spectra of ZnS. Further, the peak at 161.7 eV is assigned to sulfur, S 2p [23], and the peak at 530 eV that is corresponding to oxygen O 1s. No other unknown peaks were observed on the survey spectra of the ZnS that reflected the purity of the synthesized sample. Upon the Ni incorporation on the ZnS, the XP characteristic peaks of ZnS such as Zn 2p, S 2p, and O 1s can be observed on the survey spectra of the Zn_0.98_Ni_0.02_S and Zn_0.92_Ni_0.08_S samples. Further, one can hardly observe the Ni 2p peak at 855 eV as seen on the inset of Figure 5a. The low intensity of the Ni 2p peak is justified with the lower content as well as the fact that XPS is a surface technique. Figure 5b shows the Zn 2p high-resolution spectra of the examined samples, clearly no peak shifting, and the Zn 2p doublet positioned at 1044.3 and 1021.2 eV with an energy difference of 23.1 eV that confirm the cubic phase of ZnS [24]. Figure 5c depicts the high-resolution spectra of O 1s for the ZnS sample, which deconvolve into two peaks at 529.8 eV corresponding to the Zn–O bond and at 531.2 eV corresponding to oxygen deficiencies within the ZnS matrix. The O 1s spectrum from the Zn_0.92_Ni_0.08_S sample resembles that of the pristine ZnS, implying no changes in the oxygen component upon incorporation of the Ni into the ZnS matrix.

### 3.6. UV–Vis Absorption Measurements

**Figure 6:**
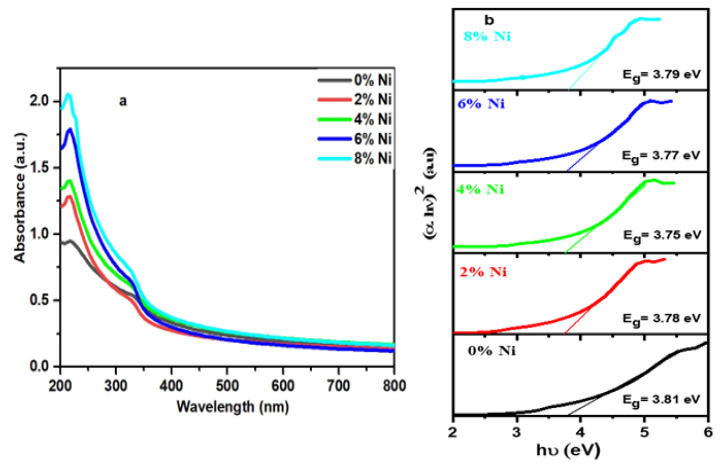
Optical absorption curves: (**a**) absorbance vs. wavelength, (**b**) graphs of (αhυ)^2^ vs. hυ for all samples.

Figure 6 displays the UV–vis absorption spectra (a) and Tauc plot of (*α*h*υ*)^2^ vs. h*υ* (b) for the Zn_1−*x*_Ni_*x*_S samples with varying values of *x* (0, 0.02, 0.02, 0.06, and 0.08).

One of the most crucial characteristics of any material for a variety of industrial applications is its optical quality. At room temperature, the UV–vis absorption spectra of the Zn_1−*x*_Ni_*x*_S samples with various compositions (*x* = 0, 0.02, 0.04, 0.06, and 0.08) were measured. The measurements were taken in the wavelength range of 200−800 nm. Figure 5 illustrates that all the Zn_1−*x*_Ni_*x*_S samples display a prominent absorption band in the UV region, specifically within the range of 250–300 nm. However, these samples exhibit weak absorption in the visible region. The progressive replacement of Ni^2+^ in the ZnS lattice leads to variations in the absorption coefficient of the sample and affects the location of the absorption peak. The band gap may be estimated utilizing the Tauc relation based on the UV–vis measurements [25,26,27,28].

(αh*ν*)^2^ = B (h*ν* − E_g_),

where the absorption coefficient α, input photon energy h*v*, Plank’s constant h, band gap energy Eg, and a constant B, and *α* represent the absorption coefficient that may be determined using this equation [29].

α = 2.303 (A/d)

where A represents the optical absorbance, whereas d represents the thickness.

By plotting the relationship between (αh*v*)^2^ and the photon energy (h*v*) and analyzing the linear portions of the resultant curves, it is possible to derive the values of E_g_ [30], as seen in Figure 5b. The optical band gap energy values for the Zn_1−*x*_Ni_*x*_S (*x* = 0, 0.02, 0.04, 0.06, and 0.08) samples are measured to be 3.81, 3.78, 3.75, 3.77, and 3.79 eV, respectively. All the Zn_1−*x*_Ni_*x*_S samples had direct band gap values significantly higher than the bulk ZnS value of 3.67 eV, which resulted in a blueshift phenomenon. The observed blueshift in the optical band gap of the Ni-doped ZnS samples can be explained by both quantum confinement effects and the formation of Ni-induced states within the band structure. As the Ni concentration increases from 0% to 8%, the optical band gap values of Zn_1−_*_x_*Ni*_x_*S (*x* = 0, 0.02, 0.04, 0.06, and 0.08) vary from 3.81 eV to 3.79 eV, remaining higher than the bulk ZnS value of 3.67 eV. This increase in the band gap energy indicates a shift, which is traditionally attributed to quantum confinement effects. When the particle size is reduced to the nanoscale, the energy gap widens, causing absorption to occur at shorter wavelengths. However, beyond quantum confinement, the blueshift in Ni-doped ZnS samples can also be influenced by the introduction of Ni-induced states. The integration of Ni^2+^ into the lattice of ZnS leads to localized electronic states near the conduction band, altering the band structure and resulting in an increase in the effective band gap. The lattice distortion caused by Ni doping, as evidenced by the observed changes in interplanar spacing, also contributes to the shift. The smaller particle size and higher surface tension associated with Ni doping further distort the lattice structure, reducing the lattice constants and shortening the bond lengths. This combination of quantum confinement, Ni-induced electronic states, and lattice distortion effectively explains the blueshift observed in the optical properties of the Ni-doped ZnS samples [31,32,33]. In addition, Figure 6b shows the impact of different concentrations of Ni doping on the band gap energy of the Zn_1−*x*_Ni_*x*_S samples as the Ni-doped ZnS samples have a lower band gap than the undoped samples. The band gap gradually decreases as the concentration of Ni doping increases, reaching a minimum value of 3.75 eV for a 4% Ni concentration. Nevertheless, when the concentration of Ni exceeds when the amount of Ni doping goes above 4%, the band gap of the Zn_1−*x*_Ni_*x*_S samples goes up a little along with the boost in the Ni content. The redshift in concentration causes a decline in the band gap, which we attribute to the sp–d spin exchange interactions. The s–d and p–d exchange interactions increase the likelihood of correcting the positive and negative potential to the conduction band and the valence band edge, respectively; this leads to a narrowing of the band gap [34,35]. The Burstein–Moss shift phenomenon contributed to the observed increase in the band gap (blueshift) at high doping levels (6% to 8% Ni). of Ni ions reaches a threshold, resulting in an increase in the number of carriers contributed by these ions [36]. An increase in the carrier density causes the Fermi level to shift towards the conduction band, whereas changes in the transition levels cause the energy gap to expand.

### 3.7. Energy Transfer Mechanism

When Ni is doped into ZnS nanoparticles, Ni^2^⁺ ions typically replace Zn^2^⁺ ions in the ZnS lattice due to their similar ionic radii, a process known as substitutional doping. This substitution creates localized defect sites in the crystal lattice, altering the electronic environment of the ZnS host and acting as trapping sites for charge carriers, such as electrons and holes, generated during excitation. The incorporation of Ni^2^⁺ ions introduces new energy states within the band gap of ZnS, associated with the 3d orbitals of Ni^2^⁺. These states, positioned below the conduction band of ZnS, depend on the local environment and Ni concentration. Upon exposure to light or other forms of excitation, electrons in the ZnS valence band absorb energy and transition to the conduction band. However, in the presence of Ni^2^⁺, some electrons may be trapped in these intermediate energy states rather than reaching the conduction band, leading to non-radiative relaxation processes and a reduction in overall energy. The energy transfer mechanism in Ni-doped ZnS involves several pathways. Resonance energy transfer occurs non-radiatively from the excited ZnS lattice to Ni^2^⁺ ions through dipole–dipole interactions, resulting in photoluminescence quenching and reduced radiative recombination efficiency. Additionally, some excess energy is dissipated as heat via phonon interactions due to local lattice distortions around the Ni^2^⁺ sites, influencing the crystal structure and size of the nanoparticles. Electrons trapped in Ni-induced states may recombine with holes from the valence band, emitting lower-energy photons or undergoing non-radiative relaxation. These energy transfer and thermalization processes can cause localized heating effects, promoting atomic diffusion and potentially impacting the growth kinetics of ZnS nanoparticles. Initially, as the Ni content increases, these processes may enhance particle growth due to increased defect sites and energy dissipation. However, beyond a certain concentration, such as 6% Ni doping, the aggregation of Ni dopants may induce strain or disrupt the crystal lattice, causing a decrease in particle size due to lattice contraction or the formation of amorphous regions [12,13]. In conclusion, Ni doping in ZnS nanoparticles introduces localized energy states that facilitate energy transfer processes, such as resonance energy transfer and non-radiative relaxation. These processes significantly affect the photophysical properties, size, and stability of ZnS nanoparticles.

The results presented in Table 3 highlight the advantages of the current work over previous studies in several aspects. Firstly, the ZnS nanomaterials synthesized in this study using the hydrothermal method demonstrated an energy band gap of 3.81 eV for undoped ZnS and 3.75 eV for Ni-doped ZnS. These values are comparable to or narrower than those reported for ZnS prepared by other methods, such as co-precipitation or ultrasonic spraying, indicating that the hydrothermal method employed in this study is effective in tailoring the band gap energy. Moreover, the band gap narrowing observed in the Ni-doped ZnS (3.75 eV) compared to undoped ZnS (3.81 eV) demonstrates a successful modification of the electronic structure, which is crucial for enhancing the photocatalytic or optoelectronic properties. Furthermore, the present work shows that the energy band gaps of 3.81 eV and 3.75 eV, respectively, are significantly higher than the standard P25 reference material with an energy band gap of 3.15 eV [37]. This tuning of the band gap is advantageous for specific applications, such as photocatalysis and energy conversion, where a precise band gap is essential for optimal performance. Additionally, the hydrothermal method used in this study offers a more controlled synthesis environment, potentially resulting in better crystallinity and uniformity of the nanoparticles. Unlike co-precipitation or ultrasonic spraying methods, which may produce particles with more variability in size and shape, the hydrothermal method can provide a more uniform distribution of dopants, leading to more consistent and reproducible material properties. In summary, the advantages of the current work lie in the effective band gap tuning through Ni doping, the use of a hydrothermal method that allows for better control over particle characteristics, and the production of materials with potential applicability in energy-related fields, such as photocatalysis, where precise control of the band gap and material properties is critical.

## 4. Conclusions

This study successfully synthesized both undoped and Ni-doped ZnS nanoparticles via a hydrothermal method, providing a detailed characterization of their structural, optical, and surface properties. The XRD analysis confirmed the cubic crystal structure of ZnS, with the successful incorporation of Ni ions without altering the overall lattice configuration. The incorporation of Ni ions led to changes in the average crystal size, with an increase up to 6% Ni doping and a subsequent decrease at higher concentrations. FT-IR spectroscopy validated the presence of characteristic Zn–S bonds and indicated successful Ni incorporation through shifts and intensity variations in the vibrational bands. SEM and EDX analyses revealed the homogeneous distribution of Ni within the ZnS matrix, while XPS further confirmed the chemical composition and bonding states of the elements. UV–vis spectroscopy demonstrated a blueshift in the band gap with varying Ni concentrations, highlighting changes in electronic transitions attributed to Ni doping. Moreover, the modification of ZnS nanoparticles with Ni significantly adjusts the surface area and pore characteristics, contributing to the improved photocatalytic efficiency observed in this study. These findings illustrate how Ni doping can effectively tune the properties of ZnS nanoparticles, making them promising candidates for applications in photocatalysis, optoelectronics, and other fields requiring specific optical and electronic properties. Further exploration could focus on optimizing the Ni doping level to enhance the desired properties for targeted applications.

## Figures and Tables

**Table 1:** Summarize miller indices, interplanar spacing, average lattice constant, and average crystal size of ZnS nanoparticles and Ni-doped ZnS.

Ni %	hkl	d_hkl_	Average Lattice Constant a	Average Crystal Size
0 (ZnS)	111	3.09257	5.364	5.27 nm
	220	1.89537		
	311	1.62041		
2	111	3.08734	5.356	5.45 nm
	220	1.89301		
	311	1.61831		
4	111	3.07986	5.358	5.83 nm
	220	1.89571		
	311	1.62147		
6	111	3.0762	5.351	5.71 nm
	220	1.89377		
	311	1.61852		
8	111	3.10657	5.386	5.60 nm
	220	1.90367		
	311	1.62588		

**Table 2:** Surface area and pore characteristics of ZnS nanoparticles and Ni-doped ZnS.

Materials	S_BET_/m^2^ g^−1^	D_BJH_/Å	V_BJH_/cm^3^ g^−1^
0% Ni (ZnS)	52.66	26.73	0.2804
2% Ni	33.78	20.15	0.2287
4% Ni	41.49	21.54	0.2931
6% Ni	44.45	20.20	0.3014
8% Ni	42.94	21.59	0.2637

**Table 3:** Presents a comparison of the preparation methods and different dopants affecting the energy band gap of ZnS, as reported in the literature, in relation to the ZnS synthesized in this study.

Materials	Preparation Method	Energy Band Gap (eV)	References
ZnS	Co-precipitation	3.96	[38]
ZnS:Mn		4.07	[38]
ZnS:Cu		3.92	[38]
Zn_0.9_Al_0.1_S	Co-precipitation	4.01	[39]
Zn_0.92_Al_0.08_S		3.94	[39]
Zn_0.97_Cr_0.03_S	Co-precipitation	4.03	[16]
ZnS	Co-precipitation	3.8	[40]
Ni-doped ZnS		3.7	[40]
ZnS:Br (2%)	Ultrasonic spraying	3.8	[41]
P25	Standard	3.15	[37]
ZnS	Hydrothermal	3.81	This Work
Zn_0.96_Ni_0.04_S	Hydrothermal	3.75	

## Data Availability

On reasonable request, the author will make the datasets created during and/or analyzed during the current investigation.
